# Mediators of Physical Activity on Neurocognitive Function: A Review at Multiple Levels of Analysis

**DOI:** 10.3389/fnhum.2016.00626

**Published:** 2016-12-08

**Authors:** Chelsea M. Stillman, Jamie Cohen, Morgan E. Lehman, Kirk I. Erickson

**Affiliations:** ^1^Department of Psychiatry, School of Medicine, University of Pittsburgh Medical Center, PittsburghPA, USA; ^2^Center for the Neural Basis of Cognition, University of Pittsburgh, PittsburghPA, USA; ^3^Department of Psychology, University of Pittsburgh, PittsburghPA, USA

**Keywords:** brain, cognition, exercise, physical activity, mechanisms, mediation

## Abstract

Physical activity (PA) is known to maintain and improve neurocognitive health. However, there is still a poor understanding of the mechanisms by which PA exerts its effects on the brain and cognition in humans. Many of the most widely discussed mechanisms of PA are molecular and cellular and arise from animal models. While information about basic cellular and molecular mechanisms is an important foundation from which to build our understanding of how PA promotes cognitive health in humans, there are other pathways that could play a role in this relationship. For example, PA-induced changes to cellular and molecular pathways likely initiate changes to macroscopic properties of the brain and/or to behavior that in turn influence cognition. The present review uses a more macroscopic lens to identify potential brain and behavioral/socioemotional mediators of the association between PA and cognitive function. We first summarize what is known regarding cellular and molecular mechanisms, and then devote the remainder of the review to discussing evidence for brain systems and behavioral/socioemotional pathways by which PA influences cognition. It is our hope that discussing mechanisms at multiple levels of analysis will stimulate the field to examine both brain and behavioral mediators. Doing so is important, as it could lead to a more complete characterization of the processes by which PA influences neurocognitive function, as well as a greater variety of targets for modifying neurocognitive function in clinical contexts.

## Introduction

Physical activity (PA) is important for maintaining *physical* health. PA has also been shown to maintain and improve *neurocognitive* health, but we know much less about the mechanisms by which it exerts its salutary effects on brain and cognition in humans (e.g., see Tomporowski and Ellis, 1986; Hillman et al., 2008; Gomez-Pinilla and Hillman, 2013 for reviews). However, research has found that physical *inactivity* is a risk factor for cognitive impairment, which has stimulated interest in examining whether PA can act to improve neurocognitive function, as well as the mechanisms by which it might work.

What are the cellular and molecular, brain systems, and behavioral mechanisms by which PA influences cognitive function, and how can they be identified? There are two frameworks that are typically used for causal inference—one is related to the design of the study, and the other is related to the statistical approach used for analysis (Imai et al., 2011). The gold standard for assessing causality is through an **experimental manipulation**. A study is “experimental” when an independent variable (i.e., PA) is manipulated to examine its influence on a dependent variable (i.e., cognition). In the context of exercise studies, human or animal subjects are randomly assigned to an experimental condition (i.e., exercise treatment) or control group (i.e., standard care), and the outcome of interest is assessed in each group while all other factors are held constant. In the context of PA, this type of causal evidence comes almost exclusively from *exercise training* studies when studying humans, or from animal models of exercise in which one group of animals is permitted to exercise while another group is treated as a control. Causality is established if the outcome variable (e.g., cognition) changes to a greater extent in the treatment (e.g., exercise training) group relative to the control group.

Of course, it is not always feasible or practical to randomly assign participants to groups and experimentally manipulate treatment variables such as exercise, especially when studying humans. In this case, an alternative framework for causal inference can be used. **Statistical mediation** is used to test the plausibility of causal models not only in experimental studies, but also in observational, longitudinal, or quasi-experimental designs in which random assignment did not occur and/or the treatment variable of interest was not directly manipulated. Further, statistical mediation can be used in at least two different contexts. It is important to distinguish between these contexts as they influence the type of conclusions that can be drawn from the model’s results.

In the first context (i.e., observational, longitudinal, and quasi-experimental studies), mediation models allow us to evaluate alternative causal mechanisms between the treatment and outcome variables by examining the roles of several intermediate variables that lie in the causal path. The benefit of using statistical mediation models in this context is that we are able to obtain evidence of a potential causal pathway using data or designs that are inherently non-experimental. Importantly, however, it is still not possible to rule out whether some third, confounding factor is driving the pattern of statistical mediation observed in a non-experimental design.

In the second context, a “gold standard” experimental design is used alongside a statistical mediation model. Using statistical models in experimental designs allows us to further examine intermediate factors that might covary with the treatment and outcome variables, and to test their plausibility as causal paths. Significant statistical mediation in this latter context provides more definitive evidence of a causal path because random assignment minimizes the influence of confounders and the direction of the relationships in the model can be established (i.e., it is possible to demonstrate that a manipulation caused changes in the mediator and outcome, rather than the reverse).

In both contexts described above where statistical mediation models can be used, the intermediate variable is considered a *mediator* if the coefficient describing the strength of the treatment-outcome relationship through the mediating variable (i.e., the indirect effect) is statistically significant (Preacher and Hayes, 2008). In other words, the significance of the indirect effects examines whether the mediator is a viable mechanism by which the independent (i.e., treatment) variable influences the outcome. Although statistical mediation can, and perhaps should, routinely be used in experimental studies, this approach is most frequently used to infer possible causal relationships from non-experimental data.

For the purposes of the present review, both types of causal evidence—design-related and statistical mediation—are relevant and will be discussed in the context of understanding mechanisms by which PA influences cognitive function. However, a critical caveat of the statistical mediation approach in quasi-experimental and observational studies is that causal relationships between a predictor and outcome through the mediator cannot be definitively determined, even in the case of significant statistical mediation. This is because random assignment did not occur, thus there could be pre-existing differences between the groups. However, statistical mediation approaches *can* provide evidence that one mediation pattern is more plausible than another, and they provide valuable theoretical insight for the design of future experimental studies.

Based on the description of causality outlined above, mechanisms can be conceived at multiple levels. However, most studies have taken a reductionist approach to mechanisms. To date, for example, most systematic reviews focusing on the causal mechanisms by which PA affects cognition have focused on cellular and molecular mechanisms, hereafter referred to as those at *Level 1 of analysis*. Consequently, there is a wealth of evidence (the majority from animal models) indicating that PA likely improves cognition by promoting various cellular and molecular pathways, including those responsible for neurogenesis and angiogenesis (van Praag et al., 2005), while decreasing others, such as inflammation (e.g., Parachikova et al., 2008). While information about basic cellular and molecular mechanisms is an important foundation from which to build our understanding of how PA promotes cognitive health in humans, there are other possible ways of thinking about mechanisms.

**FIGURE 1:**
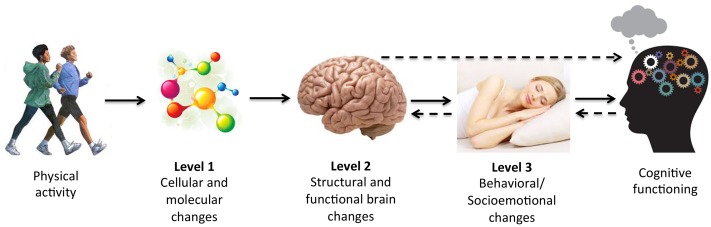
**Conceptual model of mechanisms of physical activity (PA) at multiple levels of analysis.** There are various possible mediating pathways and bidirectional effects. Several possibilities are denoted with dotted arrows.

Just as mechanisms can be assessed in multiple ways (e.g., statistically or experimentally), they can also be assessed at multiple levels, ranging from the cellular and molecular level (i.e., Level 1) up to other, more macroscopic levels of analysis (see **Figure 1** for conceptual model). We will refer to brain systems and behavioral mechanisms as those at *Level 2* and *Level 3 of analysis*, respectively, and describe them further below. Separating mediators into different levels of analysis may be informative because PA-induced changes to cellular and molecular pathways (Level 1) likely initiate changes to macroscopic properties of the brain (Level 2) and behavior (Level 3) that in turn influence cognition. For example, changes in brain structure and function (Level 2) and behaviors, such as in socioemotional functions (e.g., mood, motivation, or sleep) (Level 3) could mediate improvements in cognitive performance following PA. Crucially, our choice to organize potential mediators into different levels is not meant to imply that the mechanisms at each level are mutually exclusive. In fact, pathways identified using Levels 2 and 3 analyses are necessarily invoked by changes at lower levels of analysis, and bidirectional effects likely exist between levels (e.g., feedback loops from higher levels influencing lower levels of analysis). For conceptual purposes, however, we have chosen to discuss evidence for mediators at each level of analysis separately.

Prior reviews have focused almost entirely on Level 1 mechanisms at the expense of the other levels. The goal of this review is to use a more macroscopic lens that attempts to identify Levels 2 and 3 mediators of the association between PA and cognitive function. To accomplish this, we will briefly summarize what is known regarding cellular and molecular mechanisms and will devote the remainder of the review to discussing the brain systems and behavioral/socioemotional mechanisms at Levels 2 and 3 of analysis. It is our hope that discussing mechanisms at multiple levels of analysis will stimulate the field to examine systems and socioemotional mediators, leading to a more complete characterization of the processes by which PA influences neurocognitive function, and a greater variety of targets for modifying neurocognitive function.

In summary, the main aim of the present review is to summarize the evidence that macroscopic brain and behavioral/psychological changes can be mediators of the effects of exercise. We define high quality evidence of mediation as either coming from randomized controlled trials (RCT) or from correlational/cross-sectional work in which a statistical mediation model is tested after finding a pattern of correlations consistent with mediation. We are not aware of any other reviews to date considering such macroscopic (i.e., non-molecular) brain and behavior changes as mediators of exercise.

### Key Definitions

Before we discuss the evidence for mechanisms at each level of analysis, we will first define several terms related to cognition and PA, as they will be used throughout the following sections. *Neurocognition* is a broad term referring broadly to the brain and its cognitive functions. We will use this term when referring to general observations about the effects of PA on both brain and cognitive outcomes. *Cognition* is a slightly more specific term. We will use this when referring to particular behavioral performance measures, such as those frequently used to assess the effects of PA in experimental studies.

*Physical activity (PA)* is a broad term referring to an activity that raises heart rate above resting levels (Caspersen et al., 1985). This could include anything from housework or gardening, to walking or lifting weights. Importantly, the term PA has also been used to refer to coordinative activities, such as those requiring balance and higher-order cognitive processes (Voelcker-Rehage and Niemann, 2013). However, it is the former definition of PA—that referring to aerobic, heart-rate-raising activity–that will be the focus of the present review as the PA field in the context of neurocognitive function is dominated by research using this definition. Further, different types of PA may have overlapping and distinct mechanisms (Voelcker-Rehage et al., 2010; Voelcker-Rehage and Niemann, 2013; Niemann et al., 2014). Aerobic PA, hereafter referred to as “PA,” is often assessed subjectively in research studies, but it can also be measured objectively using devices such as accelerometers. Regular participation in PA influences aerobic fitness. Aerobic *fitness* (hereafter just “fitness”) is a measure of cardiovascular efficiency and is often measured with a graded maximal exercise test. The main outcome measure from graded exercise tests is VO_2max_, a metric describing a person’s maximal oxygen uptake. VO_2max_ is widely accepted as the gold standard of the functional limit of the cardiovascular system. As such, it is often used as the primary objective outcome of fitness in non-experimental studies, or to assess whether a PA intervention was effective. Both PA and fitness are often used to describe the results of non-experimental designs. *Exercise* refers to any structured activity that is intended to improve physical fitness. This term is often used to describe the type of PA occurring in training studies. For the purposes of this review, we will use *PA* or *fitness* when referring to study outcomes and will use PA when discussing overall concepts or discussing mechanisms more generally. We will use *exercise* exclusively when referring to studies employing a randomized, controlled experimental design.

## Level 1: Molecular and Cellular Mechanisms

### PA and Cognitive Functioning

Much of what is known about the cellular and molecular mechanisms linking PA to cognitive functioning comes from animal models. This is because animals (most often mice or rats) can be randomly assigned to an exercise or control group while the external environments are controlled for the duration of the study. This experimental manipulation examines whether outcomes between groups can be attributed to the exercise and not to other unmeasured factors. In a typical study, animals in the exercise group are given free access to a running wheel (i.e., voluntary exercise), while those in the control group are not given access to a running wheel to ensure they are comparatively inactive; all other environmental conditions (e.g., diet) are held constant. Cognitive function is assessed at the conclusion of the study (typically lasting 2–8 weeks), and physiological and brain changes are evaluated shortly after during autopsy.

Experimental studies employing animal models have established that PA (in particular, aerobic exercise) improves cognitive function, especially in cognitive domains dependent on the hippocampus, such as spatial or relational learning and memory, object recognition (e.g., Hopkins and Bucci, 2010; Bechara and Kelly, 2013), and avoidance learning (e.g., Baruch et al., 2004; Chen et al., 2008) (see van Praag, 2008 for review). In addition, exercise increases long-term potentiation, a cellular analog of learning and memory, in a hippocampal sub-region known as the dentate gyrus (e.g., van Praag et al., 1999). Animal models have been critical in establishing that the changes initiated by exercise extend beyond behavior into cognition, prompting further research into the mechanisms underlying exercise-induced synaptic, and downstream cognitive, changes.

### Molecular Mechanisms

**FIGURE 2:**
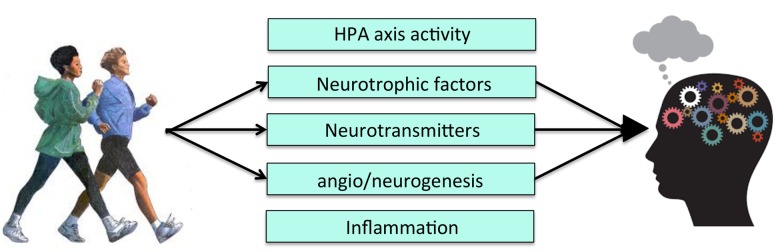
**Level 1 of analysis: Possible molecular and cellular mechanisms of PA.** HPA, Hypothalamic-pituitary-adrenal axis activity.

Exercise exerts its salutary effects on learning and memory by modulating key growth factor cascades responsible for energy maintenance and synaptic plasticity (**Figure 2**). Currently, the two pathways most studied in relation to the PA-neurocognition link are brain-derived neurotropic factor (BDNF) and insulin-like growth factor-1 (IGF-1) (for reviews of mechanisms see Cotman et al., 2007; Gomez-Pinilla and Hillman, 2013). Exercise increases BDNF and IGF-1 gene expression and protein levels, both in the periphery, as well as in several brain regions, with the most robust and long-lasting changes in the hippocampus (Voss et al., 2013b; Duzel et al., 2016). BDNF and IGF-1 signaling are considered to be causal pathways underlying exercise-related neurocognitive improvements because they are *necessary* to observe exercise-induced cellular effects. That is, experimentally blocking signaling in these pathways (e.g., with receptor-blocking ligands) eliminates or attenuates the beneficial effects of exercise on cellular and molecular pathways related to cognition (e.g., long-term potentiation) (Cotman et al., 2007). Most of the initial studies manipulating the action of BDNF or IGF-1 pathways have focused on the cellular consequences of this manipulation, and not on cognition itself. However, there is evidence suggesting that blocking BDNF attenuates behavioral learning and memory improvements following exercise (Vaynman et al., 2004). Therefore, exercise-related increases in at least one of these growth factors has been directly linked to both cellular and cognitive changes.

Blocking IGF-1 signaling also prevents exercise-induced increases in BDNF, suggesting that the two pathways converge at certain points in their cascades (Carro et al., 2000; Ding et al., 2006). Thus, the results of experimental animal studies have established that both BDNF and IGF-1 are mediators of exercise-induced cognitive improvements and that their relationship may be interdependent, and also involve other molecules and cascades. Therefore, while BDNF and IGF-1 are two molecular pathways affected by exercise, there are likely many others involved. Nonetheless, the heavy theoretical focus on these two molecular pathways has spurred research into other possible cellular mechanisms underlying effects of exercise on neurocognition, such as vascular endothelial growth factor (VEGF) and various neurotransmitters (e.g., serotonin) (Fabel et al., 2003; Cotman et al., 2007; Hamilton and Rhodes, 2015).

### Cellular Mechanisms

Angiogenesis, the development of new blood vessels, and neurogenesis, the development of new neurons, are complex cellular changes that result from increased growth factor production and up-regulated molecular cascades. Both processes have emerged as viable candidates mediating the relationship between exercise and cognition (Cotman et al., 2007) (**Figure 2**). Changes in neurovasculature precede neurogenesis in rodents, particularly in the hippocampus (van Praag, 2008). Therefore, improvements in cognition following exercise may be due, in part, to increased growth of blood vessels, which in turn stimulates cell proliferation and survival.

Neurogenesis, particularly that which occurs in the dentate gyrus, is one of the most replicated cellular changes linked to exercise (van Praag, 2008). The mediating role of neurogenesis to exercise-related cognitive changes was once controversial (Leuner et al., 2006; Meshi et al., 2006), but it is now more accepted as a viable mechanism underlying learning and memory improvements (Clark et al., 2008; van Praag, 2008). Following 2–3 weeks of voluntary exercise in rodents, there is an increase in dendritic length and complexity of existing neurons, as well as neural progenitor proliferation, in the dentate gyrus compared to control animals (Eadie et al., 2005; Cotman and Berchtold, 2007). Importantly, exercise-induced increases in the number of newborn, dividing neurons occurs in regions that overlap with those showing enhanced synaptic plasticity and growth factor expression following exercise (e.g., dentate gyrus). Exercise-induced changes in vasculature are less regionally specific (Cotman et al., 2007; Voss et al., 2011; Vivar et al., 2013). Moreover, abolishing the division of new neurons during environmental enrichment manipulations that include exercise, or inhibiting the integration of these neurons into the existing hippocampal cell structure, eliminates the learning and memory improvements typically observed following such manipulations (Bruel-Jungerman et al., 2005; Vivar et al., 2012). Thus, animal studies have suggested that neurogenesis and the survival and integration of new neurons into existing cellular networks are *necessary* to observe some cognitive improvements, particularly in the domain of learning and memory, following exercise. Yet, several studies have suggested that neurogenesis alone is not enough to induce cognitive changes, and that cognitive changes only arise when the new neurons successfully integrate themselves within an existing body of cells. This pattern of results establishes neurogenesis – potentially by way of angiogenesis – as another, slightly more macroscopic mechanism at Level 1 of analysis and further highlights the importance of examining mechanisms from multiple perspectives, even within our conceptualized three levels of analysis.

The bulk of evidence for the molecular and cellular mechanisms of exercise comes from animal models. One limitation of animal models is that the results cannot always be directly extrapolated to humans. Indeed, the cellular and molecular mechanisms of exercise-induced improvements to cognitive functioning in humans (not only in regards to learning and memory, but also other cognitive domains) remain largely unknown. This is, in part, because there are limited techniques available to measure *cellular and molecular pathways* in the human brain.

Despite these limitations, animal models have been critical in establishing that molecular and cellular changes occur in response to PA, particularly in the hippocampus. They have provided evidence that changes in molecular and/or cellular pathways mediate cognitive changes, supporting that these pathways are underlying mechanisms of the PA-cognition link. We have spoken about Level 1 mechanisms in brevity above because of the multitude of reviews already published summarizing this literature (e.g., Cotman et al., 2007; Hillman et al., 2008; van Praag, 2008; Lista and Sorrentino, 2009; Gomez-Pinilla and Hillman, 2013). However, these molecular and cellular mechanisms likely invoke more macroscopic changes in the brain, which leads us to Level 2 of analysis.

## Level 2: Macroscopic Brain Systems

### Statistical Mediation – Cross-Sectional Studies

The molecular and cellular mechanisms of exercise in humans have been difficult to establish because it is not possible to experimentally manipulate or measure cellular and molecular processes in humans in the same way we do in animals – through the use of brain tissue samples. Fortunately, advances in neuroimaging have allowed us to examine, *in vivo* and non-invasively, more macroscopic effects of PA on the structure and function of brain regions and circuits. But this leads to a critical question: Do the effects of exercise and fitness on neuroimaging markers (e.g., volume) have a mediating effect on cognitive outcomes, or are they just a meaningless by-product of increased exercise?

Most studies assessing mechanisms at Level 2 of analysis have examined how gray and white matter morphology are associated with PA and, in turn, whether these associations mediate differences in cognitive performance. We use the term *morphology* as a broad way to refer to changes in brain structure, most often assessed by measuring the volume of gray and/or white matter, or white matter integrity. Separate bodies of literature have demonstrated that brain morphology relates to cognitive function (e.g., Madden et al., 2012; Zhang et al., 2015) *or* to PA (e.g., Burzynska et al., 2014; Smith et al., 2014). However, it is a comparatively new concept to test brain morphology as a mechanism through which PA or fitness influences cognition. For example, in a cross-sectional study, Erickson et al. (2009) examined the possibility that links between fitness and memory function could be accounted for by hippocampal volume. Using statistical mediation modeling, they demonstrated that hippocampal volume significantly mediated the relationship between fitness and spatial memory. The authors used a statistical framework to test their mechanistic hypothesis because the study was not an experimental manipulation of PA (i.e., a randomized controlled trial). However, the results provide insight into a possible casual role of the hippocampus that would be further tested in later experimental manipulations, as described below.

Similar statistical mediation has been reported across the lifespan, suggesting that these associations may be independent of age. For example, in a group of 49 preadolescent children, Chaddock et al. (2010) found that higher-fit children had larger hippocampal volumes compared to lower-fit children, and that larger hippocampal volumes were associated with superior relational memory performance. Importantly, they found that bilateral hippocampal volume mediated the relationship between fitness and memory task performance. A study of older adults with mild cognitive impairment (MCI) reported similar pattern of results using hippocampal volume (Makizako et al., 2015). The results of these studies are consistent with animal studies demonstrating that the cognitive-enhancing effects of exercise can be traced to changes in the molecular and cellular architecture of the hippocampus. However, one limitation from human studies is that we cannot determine which molecular and cellular pathways are mediating the associations with hippocampal volume (Braun and Jessberger, 2014).

Importantly, PA research in humans has also revealed that the hippocampus is not the only region mediating the link between exercise and cognition. Recent work suggests that changes to regions other than the hippocampus (e.g., prefrontal cortex) mediate some cognitive improvements in humans. For example, in a cross-sectional study, Weinstein et al. (2012) demonstrated that higher cardiorespiratory fitness levels were associated with better performance on both executive control and working memory tasks. Fitness levels were also associated with greater gray matter volume in several prefrontal brain regions. Further, the volume of these prefrontal regions statistically mediated the relationship between fitness and executive function and working memory performance. Similarly, Verstynen et al. (2012) found that caudate nucleus volume statistically mediated the relationship between fitness and cognitive flexibility, a function known to be supported by this region. Thus, in addition to the hippocampus, volumetric differences in the prefrontal cortex and caudate nucleus may mediate fitness- or exercise-related improvements in executive control and cognitive flexibility. These results demonstrate, as would be expected, that brain regions that support certain cognitive processes are the same regions that also statistically mediate associations between fitness or PA and cognitive performance in particular domains.

In addition to the cross-sectional studies showing that gray matter volume may statistically mediate the fitness-cognition relationship, the integrity of white matter tracts may also mediate the link between PA and cognitive functioning. The first line of evidence for this idea comes from studies showing that white matter integrity has a clear association with cognitive performance across a number of domains (Fjell et al., 2011; Bennett and Madden, 2014). The second line of evidence comes from studies showing that higher levels of PA are associated with greater white matter integrity (Smith et al., 2016). Given these patterns of findings, white matter integrity is another potential mediator of the link between PA and cognitive performance. This mechanism has only recently been tested. In two independent samples with a total of 267 healthy older adults, Oberlin et al. (2016) reported that white matter integrity in diffuse tracts statistically mediated the relationship between cardiorespiratory fitness (as measured by a VO_2max_ test) and spatial working memory performance. These tracts included those connecting the medial temporal to prefrontal cortices – the same brain regions discussed above that have been found to be associated with fitness and PA in studies examining gray matter volume. Overall, these results extend the research on brain volume by demonstrating that aerobic fitness may also be associated with cognition through its associations with white matter microstructure.

The studies described above provide evidence that changes in the structure of both gray and white matter statistically mediate the relationship between PA (or fitness, in the case of cross-sectional work) and cognition. However, PA could also induce changes to the functioning, most often operationalized as functional activation or connectivity, of certain brain regions as a result, *or independent of*, changes in brain structure. Functional MRI (fMRI) markers have been found to differ between groups or change in response to an intervention; the question is if these changes mediate improvements in cognition. Several recent, cross-sectional studies examined functional activation as a statistical mediator of the effects of PA on cognition. Building on evidence of a relationship between fitness, executive control, and prefrontal functioning (Colcombe et al., 2004), Wong et al. (2015) examined the relationship between cardiorespiratory fitness (via VO_2max_), executive functioning (via dual-task processing), and prefrontal cortex activation. A statistical mediation model revealed that activation of a region in the anterior cingulate/prefrontal cortex significantly mediated the relationship between cardiorespiratory fitness and dual task performance, such that those who were more fit had more activation in this region. Similarly, Hyodo et al. (2016) found that the level of activation in the left dorsolateral prefrontal cortex statistically mediated the association between higher fitness and less cognitive interference (via a Stroop task). A recent study reported a pattern of relationships consistent with a mediating role of prefrontal cortex activation to the fitness-executive control relationship using (functional Near Infrared Spectroscopy; fNIRS) (Albinet et al., 2014). The pattern of results reported in these cross-sectional studies supports the argument that PA influences cognition through its effects on the functional allocation of neural resources (i.e., functional activation) during cognitive tasks. The results of several other cross-sectional studies assessing links between fitness, neural functioning, and cognition provide support for this general idea (Dupuy et al., 2015; Gauthier et al., 2015). However, these studies did not fully test for mediation because they either did not observe or did not test for the prerequisite correlations amongst the variables.

Cross-sectional studies utilizing statistical mediation provide a theoretical and mechanistic foundation about the relationships between cardiorespiratory fitness, brain, and cognition. However, their correlational nature leaves open the possibility that the observed behavioral and structural fitness-related differences between high and low-fit groups are caused by some unmeasured factor. RCTs are necessary to account for potential selection bias, as well as to establish a direct, causal relationship in humans between aerobic fitness, brain structure, and cognitive functioning.

### Experimental Mediation – Randomized Controlled Trials

There have been numerous RCT examining the effects of exercise on cognition *or* on brain outcomes (Kramer et al., 2006; Hillman et al., 2008; Smith et al., 2010). However, only a small subset has examined both brain *and* cognitive outcomes in the same study, allowing for causal inference (**Table 1**). Even fewer of the existing RCTs on this topic have included both cognitive and brain changes within a statistical model in order to definitively demonstrate a mechanism of exercise at Level 2 of analysis. In fact, while many of the RCTs that will be discussed below have shown promising patterns, only one tested for statistical mediation.

**Table 1:** Evidence for mechanisms of PA at Level 2 of analysis.

Lead author	Year	Sample	Mechanistic finding	Type mediation
Albinet	2014	34 older adults (all female)	Increases in cerebral oxygen responses in the right DLPFC partially mediate relationship between aerobic fitness and executive control performance	Statistical
Chaddock	2010	49 preadolescent children	Hippocampal volume mediates relationship between aerobic fitness and relational memory	Statistical
Chaddock-Heyman	2013	23 preadolescent children	Increases in prefrontal activation and executive functioning following a 9-month aerobic exercise intervention	Experimental
Colcombe	2004	41 older adults (study 1); 29 older adults (study 2)	Greater activation in prefrontal, parietal, and anterior cingulate cortex, and well as increased cognitive control performance in higher aerobically fit (Study 1) or aerobically trained (Study 2) individuals	Statistical (Study 1); Experimental (Study 2)
Erickson	2009	165 older adults	Hippocampal volume mediates relationship between cardiorespiratory fitness and spatial memory	Statistical
Erickson	2011	120 older adults	Volume of the hippocampus and memory performance increase following a 12-month aerobic exercise intervention	Experimental
Hillman	2014	221 preadolescent children	Frontal activation, cognitive inhibition, and flexibility increases following 9-month aerobic exercise intervention	Experimental
Hyodo	2015	60 male older adults	Activation of left dorsolateral prefrontal cortex mediates relationship between fitness and cognitive control	Statistical
Kamijo	2011	43 preadolescent children	Increases in cognitively relevant frontal ERP component (CNV) and in working memory following a 9-month aerobic exercise intervention	Experimental
Kraft	2014	43 overweight children	Activity increases in the anterior cingulate cortex and decreases in precentral gyrus and parietal cortex following 8-month aerobic exercise intervention; Improved cognitive control performance	Experimental ^∗^No group × Time interactions for any neurocognitive outcome
Krogh	2014	79 clinically depressed older adults	Positive association between change in hippocampal volume and changes in memory following a 3-month aerobic exercise intervention	Experimental ^∗^Issues with adherence; no group × Time interactions
Maass	2015	40 older adults	Hippocampal volume, perfusion, and memory increases following a 3-month aerobic exercise intervention; Changes in perfusion statistically mediate relationship between volume and memory	Experimental and Statistical
Makizako	2015	310 older adults with MCI	Hippocampal volume mediates the association between PA and memory in older adults with MCI	Statistical
Oberlin	2016	241 older adults across two studies	White matter integrity mediates relationship between fitness and spatial working memory	Statistical
Pajonk	2010	24 schizophrenic patients	Hippocampal volume and short-term memory improve following 3-month aerobic exercise intervention; Changes in volume correlate with changes in memory	Experimental
Ruscheweyh	2012	62 older adults	Positive association between changes in PA and episodic memory following a 6 months mixed-intensity intervention no longer significant after accounting for the variance associated with change in anterior cingulate gray matter volume	Experimental ^∗^No group × Time interactions
Ten Brinke	2015	86 female older adults	Hippocampal volume increases following 6-month aerobic exercise intervention. Changes in volume negatively associated with changes in short-term memory	Experimental
Verstynen	2012	179 older adults	Volume of the caudate nucleus mediates relationship between fitness and cognitive flexibility	Statistical
Voss	2010	62 older adults	Connectivity of large-scale brain networks and executive functioning improve following 6-month aerobic exercise intervention; changes in connectivity correlate with changes in executive functioning.	Experimental
Voss	2013a	70 older adults	Improved fitness is associated with changes in prefrontal and temporal white matter integrity following a 12-month aerobic exercise intervention; no changes in short-term memory performance reported between groups; no mediation model could be tested	Experimental ^∗^No group × Time interaction for neurocognitive outcome
Weinstein	2012	142 older adults	Volume of prefrontal cortex mediates the relationship between cardiorespiratory fitness and working memory, as well as that between fitness and inhibitory control	Statistical
Wong	2015	128 older adults	Greater anterior cingulate cortex activation mediates relationship between fitness and dual task performance	Statistical


### Changes in Brain Structure

As in the human cross-sectional work, most RCTs examining mechanisms of PA at Level 2 of analysis have focused on its effects on brain structure, particularly on gray matter volume. In general, this literature has shown that exercise training increases brain volume particularly in the hippocampus, and these volumetric changes partially account for cognitive improvements following the intervention. In a seminal study on this topic, 120 inactive older adults were randomly assigned to a 12-month aerobic walking (experimental) group, or to a stretching and toning (control) group (Erickson et al., 2011). Following the intervention, the aerobic exercise group showed greater gray matter volume in the anterior hippocampus compared to the control group. These findings represent the first experimental evidence linking changes in exercise to changes in both gray matter volume and cognitive performance in aging humans in the context of a RCT. Further, the findings are consistent with animal models of regional specificity for the effects of exercise on the brain, such that volume changes are particularly robust in the anterior portion of the hippocampus.

One limitation of the Erickson et al. (2011) study was that they did not test whether the relation between changes in fitness levels and spatial memory could be statistically accounted for by changes in hippocampal volume. It is therefore possible that another factor associated with both changes in fitness and gray matter volume accounted for the cognitive changes. In fact, the control group in this study also showed improvements in memory performance, despite showing *decreases* in hippocampal volume, making the causal links between changes in fitness and changes in hippocampal volume and spatial memory in the experimental group tenuous. Further, only the volumes of subcortical regions were assessed; potential changes in cortical regions, such as the prefrontal cortex, were not examined (but see Colcombe et al., 2006; Ruscheweyh et al., 2011 for evidence of cortical volume changes). Nonetheless, these results are important, as they were the first experimental evidence to suggest that exercise training can increase the volume of the hippocampus and improve memory in older adults.

In addition to examining mechanisms of exercise in healthy older adults, RCTs have also examined whether exercise can increase gray matter volume and cognition in clinical samples. The patient groups studied are those in which there are well-known hippocampal deficits, including schizophrenia (Pajonk et al., 2010), major depressive disorder (Krogh et al., 2014), and MCI (Ten Brinke et al., 2015). Each of these studies reported increases in gray matter volume in the hippocampus, but results were mixed regarding whether the exercise intervention improved cognition. For example, Pajonk et al. (2010) conducted a study with 24 participants, 16 of whom had schizophrenia. Eight of the patients and all of the healthy participants (*n* = 8) were enrolled in an aerobic (cycling) exercise intervention (*n* = 16), while the other eight patients played table tennis as a low-aerobic control activity. The authors found that hippocampal volume increased and short-term memory improved in the exercise group following the intervention, but not in the non-exercising control group. Short-term memory and schizophrenic symptom severity improved with changes in hippocampal volume in schizophrenics, suggesting a possible mediating role of hippocampal changes on behavioral outcomes. Unfortunately, these associations were not tested using a statistical mediation framework (see Firth et al., 2015 for mixed findings).

Increased cerebral perfusion has also been suggested as a possible mechanism for the cognitive-enhancing effects of exercise. For example, Maass et al. (2015) combined brain volume, perfusion, and memory change outcomes from their 3-month intervention and found that changes in gray matter volume could be accounted for by changes in cerebral perfusion. These results suggest that perfusion changes may mediate the effects of exercise on both gray matter volume *and* memory performance. Thus, while gray matter volume is the most widely studied Level 2 mechanism of exercise on cognitive outcomes, there are other neuroimaging modalities tapping into different components of brain health that could shed light on the mechanisms of volumetric and cognitive changes (e.g., see Zimmerman et al., 2014).

Changes in white matter microstructure may be another mechanism for the effects of PA on cognition because white matter supports communication between brain regions. Greater PA is linked to white matter preservation (Sexton et al., 2016) and decreased white matter integrity is linked to cognitive deficits (Roberts et al., 2013). This pattern raises the possibility that white matter health is a mechanism underlying the effects of PA on cognition. In the first RCT to examine this possibility, Voss et al. (2013a) evaluated the effects of a 12-month exercise intervention on white matter integrity and cognitive performance in healthy older adults. Seventy older adults were randomly assigned to either an aerobic walking or toning/stretching group; the groups participated in their respective activities for 40 min per day, 3 days per week. Increases in temporal and prefrontal white matter integrity, assessed via fractional anisotropy (FA), and memory were positively associated with improvements in fitness. However, the exercise-induced changes in FA were not associated with changes in memory performance. One possible explanation for this null finding is that the sample size was too small, limiting the statistical power to detect a relationship between FA and memory. This is a limitation that applies to many of the RCTs conducted to date.

### Exercise-Induced Changes in Brain Function

Changes in brain function in response to exercise, as measured by fMRI, could potentially precede changes in brain structure. It is also possible that exercise induces changes in brain function that are independent of changes in structure (i.e., not simply a byproduct of structural changes). Functional imaging studies of exercise therefore offer information about another potential brain mechanism underlying cognitive changes in response to exercise. Although less studied compared to structural change, exercise-related changes in brain function have been examined in the context of several RCTs (e.g., Voss et al., 2010; Kamijo et al., 2011; Chaddock-Heyman et al., 2013; Hillman et al., 2014; Krafft et al., 2014). Unlike RCTs examining structural outcomes, however, most of the RCTs focusing on functional outcomes have focused on changes in prefrontal cortex functioning, rather than the hippocampus. For example, using fMRI, Chaddock-Heyman et al. (2013) observed that children participating in a 9-month exercise intervention, 5 days per week, showed improved executive control performance and increased prefrontal activation patterns following the intervention, similar to the pattern seen in a healthy adult comparison group. Krafft et al. (2014) reported a similar pattern of findings in obese children: Following an 8-month intervention, obese children showed improved cognitive control and increased activation in a comparable set of prefrontal brain regions to those reported by Chaddock-Heyman et al. (2013). Further, using similarly aged (i.e., childhood) samples, two additional studies by Kamijo et al. (2011) and Hillman et al. (2014) observed that exercise improved cognitive performance (i.e., working memory and cognitive flexibility, respectively) and increased frontal electrophysiological indices of cognitive preparation and flexibility (i.e., contingent negative variation and P3 amplitude, respectively) following 9-month exercise interventions. Thus, in the pre-pubescent, developing brain, exercise has been shown to improve executive functioning, and these improvements have been associated with increased activation or neural responsiveness in prefrontal brain regions.

At least two RCTs suggest that the functional changes induced by exercise extend to older adults, although perhaps in a less regionally specific manner. Using fMRI, Voss et al. (2010) showed that a 12-month walking intervention increased functional connectivity among regions within two large-scale brain networks: The default mode and the frontal executive networks (FEN). The increased functional connectivity in the FEN, a network that includes several prefrontal brain regions, was associated with improvements in executive control performance. A seminal study by Colcombe et al. (2004) reported similar findings in the functioning and recruitment of the FEN following a shorter, 6-month exercise intervention in older adults, supporting the claim that changes to large scale brain networks may occur relatively soon after the commencement of exercise training. Since large-scale brain networks are known to become less efficient and less flexible with age, these results suggest that exercise may exert more global effects on the efficiency and flexibility in which networks of brain regions interact in older adults, leading to preserved cognitive performance.

**FIGURE 3:**
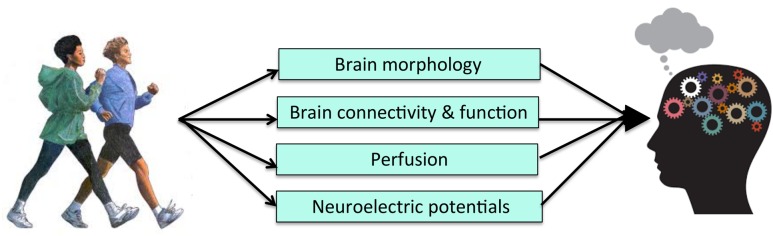
**Level 2 of Analysis: Possible brain systems-level mechanisms of PA**.

There have been a number of RCTs examining the effects of exercise on both brain outcomes and cognition. While many have demonstrated brain *or* cognitive changes following an exercise intervention, few have gone on to test for statistical mediation after finding a pattern of results consistent with a causal mechanism. Doing so, however, is important to establish the behavioral relevance of changes in neuroimaging metrics in PA-induced improvements in cognitive functioning (**Figure 3**). Further, the various differences in study design, measurement techniques, analytic approach, and study samples employed across the existing RCTs limit the mechanistic conclusions that can be drawn and highlight the need for more RCTs in this area. In particular, there is a need for RCTs to include larger samples and multiple imaging modalities in order to tease apart mechanistic questions related to temporal precedence. There is also a need to look at the activation of networks of brain regions, as well as the connectivity between them, as potential mediators. Given the recent shift in the field to focus on functional brain networks, it seems unlikely that any single brain region works in isolation to mediate cognitive improvements. More complex mediation models may prove useful for more fully capturing the mechanisms underlying the effects of PA on cognition. Nonetheless, the results of the existing body of literature are promising in that they indicate that exercise has multi-modal effects on the brain that likely underlie improvements in cognition. Further, the convergence of results, despite various differences across the studies, speaks to the robustness of the effects of exercise on both brain and cognition, and pinpoints PA as an effective tool to preserve and promote neurocognitive functioning across the lifespan.

## Level 3: Behavioral and Socioemotional Mechanisms

**FIGURE 4:**
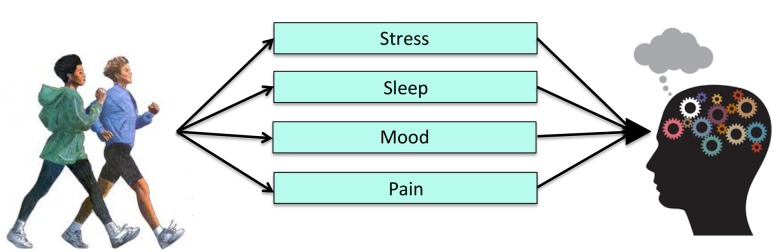
**Level 3 of analysis: Possible behavioral mechanisms of PA**.

The evidence described above implicates a number of molecular, cellular, and brain processes involved in PA, but it is likely that PA also exerts changes in other behaviors that contribute to cognitive improvements (**Figure 4**). Unfortunately, few studies have examined how mechanisms at Level 3 of analysis might underlie the effects of exercise on cognitive performance. From a clinical standpoint, however, changes in human behavior are much easier to observe and may reflect a cost-effective approach to understanding behavioral mechanisms by which exercise improves cognitive function. Thus, there is added practical value in understanding potential mechanisms at the behavioral and socioemotional level.

### Sleep as a Mediator

Sleep quantity and quality are important for healthy cognitive function (Yaffe et al., 2014). For example, both the amount and quality of sleep are considered to be important in memory consolidation and learning processes (Walker, 2009). There is also a wealth of cross-sectional and experimental evidence that supports the idea that high quality sleep leads to better performance on a variety of cognitive tasks (see Ellenbogen, 2005 for review). Sleep is therefore a critical contributor to cognitive performance.

There is also evidence that increased PA improves sleep quality. The first RCTs to examine this demonstrated that relatively brief (e.g., 10-week) exercise interventions boosted self-reported sleep quality in older adults compared to non-exercise control groups (King et al., 1997; Singh et al., 1997). These studies did not include measures of cognitive performance, so the effects of exercise-induced sleep improvements on cognition could not be determined. However, given the connections between sleep and cognition, and between sleep and PA, it is possible that PA improves cognitive outcomes by influencing sleep quality and efficiency.

The only study to date that included PA, sleep, and cognition in one model tested the hypothesis that sleep mediates the relationship between PA and executive functioning in 109 young (*n* = 59) and older adults (Wilckens et al., in press). Both PA and sleep were objectively measured with accelerometry. Wilckens et al. (in press) found that PA energy expenditure was positively associated with sleep efficiency, as well as executive functioning and processing speed. Further, sleep efficiency statistically mediated the relationship between PA and several measures of cognitive performance. Although limited by the cross-sectional nature of the design, these findings provide evidence that sleep may be a behavioral mechanism by which PA influences cognitive performance. Future RCTs in which participants are randomly assigned to multiple sleep and exercise conditions are needed to test this hypothesis directly.

### Mood as a Mediator

Low mood, often assessed using measures of depressive symptomology, is associated with poorer performance on a variety of cognitive tests (see Lichtenberg et al., 1995; Austin et al., 2001 for reviews). These cognitive deficits typically manifest in the domains of executive functioning, attention, and memory—the same domains that are most affected by PA (McClintock et al., 2010).

Increased PA is associated with improved mood, and is an efficacious approach to reduce symptoms of depression and anxiety (Byrne and Byrne, 1993; Fox, 1999; Penedo and Dahn, 2005; Ströhle, 2009; see for review Bridle et al., 2012). In fact, PA is increasingly being used as an adjunct treatment for clinically significant depression (Mead et al., 2008). Interestingly, there is also evidence that the relationship between PA and mood is bidirectional, such that poorer mood may lead to decreased PA (Roshanaei-Moghaddam et al., 2009).

Despite the fact that mood has been linked both to PA and cognitive function, only a handful of studies have considered mood as a mediating pathway through which PA influences cognition. For example, Vance et al. (2005) and Robitaille et al. (2014) both used statistical mediation in cross-sectional studies to examine the effects of several socioemotional factors (i.e., social support, depressive symptoms, cognitive activity) on the relationship between PA and cognitive performance. Consistent with their predictions and results from previous work, there were positive relationships between PA and tests of cognition, specifically in the domains of memory, processing speed, and visuospatial functioning. However, the two studies found disparate results when examining the behavioral predictors. In particular, Vance et al. (2005) found that physical inactivity had significant indirect associations with cognitive functioning through depressive symptomology and social support, while Robitaille et al. (2014) did not find that depression scores mediated the associations between PA and cognitive performance. Instead, they found that social support and cognitive engagement mediated the effects of PA on cognition (Robitaille et al., 2014). However, the indirect effects of these factors varied by cognitive outcome, suggesting that the mechanisms may vary across cognitive domains.

Unfortunately, the results of RCTs have not provided any more clarity regarding the role of factors, such as mood, on the PA-cognition relationship. One RCT examined mood as a mediating pathway with 64 younger adults randomly assigned to an exercise or control group (Lichtman and Poser, 1983). Following the intervention, the exercise group performed significantly better on an executive control test (i.e., a Stroop task) compared to the control group. The exercise group also showed a significant decrease in their depression and anxiety symptoms compared to baseline. However, the control group also exhibited decreases in depression and anxiety. Albinet et al. (2016) found similar results in a sample of 36 older adults who either participated in aerobic exercise or stretching control intervention for 21 weeks (Albinet et al., 2016). While both groups showed improvement in self-reported depressive symptomology, only the exercise group showed neurocognitive improvements. This pattern of results indicates that exercise was not the primary mechanism for the mood or cognitive improvements.

There are also studies in which mood changes following an exercise RCT, but no cognitive effects are observed. Another early RCT examined changes in psychological (including mood) and neuropsychological functioning following a 4-month aerobic exercise intervention in older adults (Blumenthal et al., 1989). Participants across three randomized groups (i.e., aerobic exercise, yoga and flexibility control, or waitlist control) did not show any clear pattern of differences on neuropsychological tests following the intervention. However, males in the aerobic exercise group showed a significant decline in depression and anxiety scores compared the control groups. There was still no clear pattern of cognitive improvements following exercise, even after training was extended for up to 10 months (Madden et al., 1989; Blumenthal et al., 1991).

Conversely, there are RCTs in which neurocognition changes, but mood does not. For example, Williams and Lord (1997) conducted an RCT examining the effects of a 42-week exercise intervention on mood and cognitive functioning in a group of older, community-dwelling women. Following the intervention, the exercise group performed better than the control group on measures of memory and processing speed. In addition, within the exercise group, individuals who reported higher baseline levels of anxiety and depression showed greater improvements in cognitive performance compared to individuals in the exercise group who reported lower baseline levels. However, as with Lichtman and Poser (1983), there were no group differences in mood symptoms following the intervention, and so exercise may not be driving the cognitive changes they observed. While none of these correlational studies were conclusive regarding whether mood mediates exercise-induced cognitive improvements, the results provide evidence that the relationship between exercise, cognition, and mood merits further exploration.

Testing the influence of exercise on cognition in clinical populations has not been as common in the literature. In a RCT of 73 older adults with Chronic Obstructive Pulmonary Disease (COPD), Emery et al. (1998) examined the effects of exercise, education about COPD, and stress management on psychological and cognitive functioning. Participants were randomized to three groups, only one of which had an exercise component (in addition to stress management and education). Post-intervention analyses revealed that the group receiving exercise (hereafter referred to as the “exercise group”) showed lower depressive symptomatology compared to their baseline scores and to the post-intervention scores of the education-only group. Participants in the exercise group also showed improved verbal fluency scores, while the other two groups’ scores did not change from their pre-intervention levels. While Emery et al. (1998) did not directly test a mediation model in this RCT, the fact that only the exercise group showed improvements in both mood and cognitive performance suggests a possible mediating link between exercise, changes in mood, and changes in cognition. However, it is difficult to interpret the exact nature of these effects because there was no group that *only* engaged in exercise. Thus, it is unclear whether the mood and cognitive improvements were due to exercise alone or to some synergistic effect of exercise, education, and stress management. Further, given that these results—the only experimental evidence to date to show both mood and cognitive changes in an exercising group—were reported in a clinical sample with known disturbances in mood (Maurer et al., 2008) and cognitive functioning (Liesker et al., 2004), it remains an open question whether the same mechanisms would underlie exercise-related changes to cognition in healthy populations. These results of the studies of PA and mood reviewed indicate that we do not yet understand whether, to what extent, and how changes to mood mediate the effects of PA on cognition.

## Discussion

The main motivation for writing the present review was to highlight the basic idea that mechanisms of PA on cognitive outcomes can be conceptualized on multiple levels, and it is possible to examine them using a variety of study designs. Historically, the discussion of mechanisms of PA or exercise on cognitive outcomes has been limited to Level 1 of analysis. There have been numerous reviews in the past decade that have described in detail the molecular and cellular mechanisms of PA. Since excellent evidence for these mechanisms already exists, we did not explain them in detail. Instead, we focused on evidence for mechanisms of PA on cognition at Level 2 and took stock of the (limited) evidence for mechanisms of PA on cognition at Level 3 of analysis.

Mechanisms at Level 2 (i.e., structural and functional brain changes) are only just beginning to be discussed in the scientific literature. Our review suggests that regional gray matter volume statistically mediates the relationship between cardiorespiratory fitness or PA and cognitive functioning, but most of these studies have been limited to cross-sectional designs. In addition, white matter microstructure and functional brain activity may also be mediating associations between fitness or PA and cognition (e.g., Wong et al., 2015; Oberlin et al., 2016). Across the studies, brain changes do not occur equally or uniformly throughout the brain; rather, they seem specific to several brain regions in particular, namely, the hippocampus and prefrontal cortex. The regional specificity of PA-related structural and functional brain changes is important because it mirrors some of the regional specificity observed in animal models (i.e., hippocampus).

Consistent with the cross-sectional work, RCTs also support the argument that changes in brain structure and function may be mechanisms underlying the relationship between PA and cognitive performance. Specifically, the majority of RCTs have reported changes in brain structure or function, as well as in cognition following the exercise intervention. However, of the 13 RCTs including both cognitive and neuroimaging measures conducted to date, only 1 has used a statistical mediation model. Thus, it has not been possible in the majority of RCTs to rule out the possibility that another, unmeasured factor that covaries with both the treatment and outcome is underlying the intervention effects observed in the brain and/or cognitive performance.

The search for mechanisms at Level 2 is further complicated for several reasons. First, while the volume or function of specific brain regions (again, mostly the hippocampus and prefrontal cortex) consistently change following exercise interventions, the evidence has been less consistent with regard to cognitive performance. For example, although the majority of RCTs discussed above report cognitive changes that are exclusive to the exercise group following training, several reported cognitive improvements in both the exercise *and* control groups—i.e., a lack of group-by-time interaction (Erickson et al., 2011; Ruscheweyh et al., 2011; Voss et al., 2013a; Krafft et al., 2014). This makes it difficult to link exercise and brain changes exclusively to the cognitive changes observed. It also highlights a key limitation of RCTs in humans: It is extremely difficult to control the behavior of participants outside of the context of the RCT. Thus, even using this gold standard design, extra-training behaviors (e.g., those in the “control” condition might inadvertently increase their PA) could lead to unexpected effects. Second, there is variability in the design of the existing RCTs in terms of, for example, activity level/engagement of the control group, intervention length, type and frequency of PA, adherence, exclusionary criteria, neurocognitive outcomes assessed, and analytic techniques. It is therefore difficult to know whether null findings are the result of this inter-study variability or a true lack of effect. Finally, there are many factors that may moderate the effects of PA on neurocognition. Despite the favorable effects of PA and cardiorespiratory fitness on brain health and cognitive function reviewed above, there is significant inter-individual variability within studies regarding the extent to which any one individual will reap the physical and cognitive benefits of PA. Thus, it is likely that mediators are being moderated by other factors, such as the presence of pathology, age, genotype, gender, and diet (see Leckie et al., 2012 for review). However, the convergence of the effects of PA on brain health, despite this wide range of variability, speaks to the robustness of PA on both brain health outcomes and cognitive function.

Along these lines, if PA is thought to enhance cognition by improving brain structure and function, then eliminating PA should have the opposite effect. Examination of the effects of PA cessation has been comparatively unexplored to date. However, there have been two recent studies on this topic that support this idea (Alfini et al., 2016; Thomas et al., 2016). Alfini et al. (2016), showed that cortical and hippocampal resting brain perfusion decreases following PA cessation after just 10 days in older adult athletes. In addition, Thomas et al. (2016) found that hippocampal volume gains following an exercise intervention in young-middle aged adults, were abolished following 2-weeks of exercise cessation. These results are interesting and important for the field because they support PA as the causal variable in mechanistic models (i.e., removing PA reverses the brain effects attributed to this behavior). However, Alfini et al. (2016) did not administer a full cognitive battery, thereby limiting an interpretation of their results with regard to cognition. Thomas et al. (2016) administered a brief cognitive battery, but found no change in cognition following their 6-week intervention. It was therefore not possible to thoroughly evaluate whether cognition (our outcome variable of interest) also decreased following PA cessation. Such evidence is needed, as it would further strengthen the causal role Level 2 mediators play in PA-related cognitive effects.

Mechanisms of PA on cognition at Level 3 of analysis have not been frequently considered or assessed. However, there are a handful of studies suggesting that this level may be important to consider for future studies (**Table 2**). Changes in sleep quality, for example, are linked to both cognition and to PA. However, only one study to date (Wilckens et al., in press) has combined all three variables in a statistical model to test whether sleep can account for the relationship between PA and cognitive performance—the results of this initial study suggest that it can. Similarly, mood is linked both to cognitive performance and PA. While several studies have considered mood along with other behavioral or socioemotional factors in statistical models assessing mechanisms of PA, virtually none have considered the unique or independent contribution of mood to the PA-cognition relationship.

**Table 2:** Evidence for mechanisms of PA at Level 3 of analysis.

Lead author	Year	Sample	Mechanistic finding	Type of mediation
Albinet	2016	36 older adults	Improvements in cognition following a 21-week exercise intervention are independent of improvements in mood	Experimental
Blumenthal	1989	101 older adults	A 4-month exercise intervention improves mood symptoms in males, but does not show any clear pattern of improvements in cognition	Experimental
Blumenthal	1991	101 older adults	4, 8, or 14 months of exercise training does not improve cognitive task performance	Experimental
Emery	1998	73 Chronic Obstructive Pulmonary Disorder patients	Exercise improves verbal fluency and reduces depressive symptoms compared to baseline scores and non-exercising groups	Experimental
King	1997	43 older adults	Improvements in self-rated sleep quality in exercising group following 16-week exercise intervention	Statistical
Lichtman	1983	64 young and mid-life adults	A 4-month exercise intervention improves performance on the Stroop Task	Statistical
Madden	1989	85 older adults and 24 younger adults	4 or 8 months of exercise does not improve cognitive task performance	Experimental
Robitaille	2014	470 older adults	Social support and cognitive engagement mediate the effects of PA on cognition	Statistical
Singh	1997	32 older adults	Exercise improves self-rated sleep quality following a 10-week intervention	Statistical
Vance	2005	158 older adults	Sedentary behavior has significant indirect associations with neurocognitive functioning through depression and social support	Statistical
Wilckens	in press	109 adults (59 younger, 50 older)	Sleep mediates the relationship between PA and executive functioning	Statistical
Williams	1997	187 older women	Exercise improves performance on tests of memory and processing speed following a 12-month intervention. Depression and anxiety symptoms decrease following the intervention for participants reporting the highest symptoms at baseline, though not differently from the non-exercising group.	Experimental ^∗^No group × Time interaction for mood outcomes


We highlighted sleep and mood as two examples of potential mechanisms at Level 3 because there are literatures linking these factors to both cognition and PA, thus making them candidate mediators. However, it is important to note that there are likely many other possible behavioral mechanisms (e.g., self-efficacy, motivation, and fatigue, pain) that should be examined in future work (e.g., see relevant reviews McAuley and Blissmer, 2000; Mullen et al., 2012; Teixeira et al., 2012). Identifying such mechanisms is important, as they would provide additional outcome targets to assess the effectiveness of PA interventions. These additional possible behavioral mechanisms were not addressed in the current review because, to our knowledge, there are no studies to date examining them as potential mediators of the PA-cognition relationship. Additional studies including both psychological and neurocognitive functioning as outcome variables are needed to enhance our understanding of this level of analysis.

The studies reviewed above suggest that the individual pieces of a model from PA to cognitive functioning through behavioral changes, such as sleep or mood, exist, but they have not been succinctly combined in one cohesive model. Future work should address the gaps in our understanding of how behavioral mechanisms modulate the effects of PA on cognition by conducting RCTs with participants aged across the lifespan and measuring both behavioral and cognitive outcomes at multiple time points. Designs with these components would allow us to test not only for experimental mediation, but also for statistical mediation following exercise interventions.

## Conclusion And Limitations

One important point of consideration/limitation for studies examining the mechanisms of PA relates to the sample size needed to test for mediation. There are a number of publications addressing this topic (Cerin et al., 2006; Fritz and Mackinnon, 2007; MacKinnon et al., 2007). These articles suggest that the sample size needed to detect mediation effects depends on the size of the effect expected and the statistical method used to test for mediation. Effect sizes generally decrease with increasing variability of the outcome and so, although not always practical, larger samples are likely needed to detect mediation effects when outcome measures are highly variable (i.e., as is often the case in neuroimaging data) (Preacher and Kelley, 2011). RCTs in which groups are made to more extremely differ on a manipulated independent variable is one possible way to decrease variability (by the making groups differ extremely) and therefore increase power to detect mediation. Thus, RCTs might be the best context in which to test for Level 2 mediators. In terms of analytical method, bootstrapping methods of testing mediation generally require smaller samples, while causal-steps approaches require the most (Cerin et al., 2006). The recommended sample size to test for a small-moderate mediation effect range from as few as *N* = 50–100 people using bootstrapping methods (e.g., Cerin et al., 2006) to anywhere from 400 to >20,000 people using more conservative causal-steps approaches (e.g., Fritz and Mackinnon, 2007). Many of the studies of PA cited above do not meet these recommended power requirements, increasing the likelihood of Types I and II error. Future studies should keep these guidelines in mind when budgeting and planning for recruitment.

Despite these considerations, there are many studies that implicate mechanisms of PA on cognition at Levels 2 and 3 of analysis that have given us the groundwork to construct our proposed mechanistic models (**Figures 2** and **3**), but very few have actually tested for statistical mediation. Doing so is critically important in order to rule out the possibility that the more macroscopic brain and socioemotional changes linked to PA are meaningless byproducts of PA. While we currently have the foundation to think about more macroscopic mechanisms that may mediate the relationship between PA and cognition, and potentially provide new, clinically relevant targets, these components need to be combined in testable models in future studies.

## Author Contributions

CS, JC, ML, and KE have seen and approved this manuscript for submission and are accountable for all aspects of the work. CS, JC, ML, and KE made substantial contributions to conceptualizing, drafting, and revising the manuscript.

## Conflict of Interest Statement

The authors declare that the research was conducted in the absence of any commercial or financial relationships that could be construed as a potential conflict of interest.
